# The PHD Transcription Factor Rum1 Regulates Morphogenesis and Aflatoxin Biosynthesis in *Aspergillus flavus*

**DOI:** 10.3390/toxins10070301

**Published:** 2018-07-20

**Authors:** Yule Hu, Guang Yang, Danping Zhang, Yaju Liu, Yu Li, Guanglan Lin, Zhiqiang Guo, Shihua Wang, Zhenhong Zhuang

**Affiliations:** 1 Fujian Key Laboratory of Pathogenic Fungi and Mycotoxins, Key Laboratory of Biopesticide and Chemical Biology of Education Ministry, and School of Life Sciences, Fujian Agriculture and Forestry University, Fuzhou 350002, China; Yule.Hu@outlook.com (Y.H.); guangyang123456@outlook.com (G.Y.); zhang1119383881@sina.com (D.Z.); yajuliu@139.com (Y.L.); yd_liyu@163.com (Y.L.); GuanglanLin@163.com (G.L.); Zhiqiang12321232@163.com (Z.G.); 2 Xiamen Genokon Medical Genokon Company, Xiamen 361115, China

**Keywords:** *Aspergillus flavus*, Rum1, conidiation, sclerotia, aflatoxin

## Abstract

*Aspergillus flavus* produces mycotoxins especially aflatoxin B_1_ and infects crops worldwide. As a PHD transcription factor, there is no report on the role of Rum1 in the virulence of *Aspergillus* spp. yet. This study explored the biological function of Rum1 in *A. flavus* through the construction of *rum1* deletion mutants and *rum1* complementation strains with the method of homologous recombination. It was found, in the study, that Rum1 negatively regulates conidiation through *abaA* and *brlA*, positively regulates sclerotia formation through *nsdC*, *nsdD*, and *sclR*, triggers aflatoxin biological synthesis, and enhances the activity of amylase. Our findings suggested that Rum1 plays a major role in the growth of mycelia, conidia, and sclerotia production along with aflatoxin biosynthesis in *A. flavus*.

## 1. Introduction

*Aspergillus flavus* is a famous opportunistic soil-borne pathogen and its harmfulness to immunosuppressed patients is only second to *A. fumigatus* [1]. *A. flavus* is the main producer of aflatoxin B_1_ (AFB_1_), which is the most toxic, tetratogenic, and carcinogenic (especially to liver) mycotoxins known [2]. IARC (the International Agency for Research on Cancer) has put AFB_1_ into Group 1 carcinogen [3]. By food contamination (including maize kernels, peanuts, cottonseeds, and tree nuts), *A. flavus* could cause diseases such as aflatoxicosis and hepatocellular carcinoma in human and animals through daily diets [1,4]. Aflatoxicosis caused by the intake of high dose of AFs could even lead to death [5]. As the most important agricultural mycotoxins producer, *A. flavus* has given rise to colossal economic losses, grain shortages, and health issues around the world [3,6]. To protect human and animal health and to allay the losses of agricultural economy, effective measures should be resorted to reduce the contamination and virulence of *A. flavus*.

The morphogenesis and secondary metabolism of pathogenic fungi are regulated by some conserved regulatory factors [7,8] in which VeA (a global regulator) regulates the production of aflatoxin by AflR in *A. flavus* and mediates sclerotia and cleistothecia formation in *A. flavus* and *A. nidulans*, respectively [9,10]. It was further identified that the heterogenous trimeric complex VelB/VeA/LaeA was involved in the connection of light-responding development and secondary metabolism regulation [11]. The master transcription factor MtfA and a VeA-dependent element involved in the regulation of ST biological synthesis in *A. nidulans* was found to take part in the AFB_1_ biosynthesis, fungal development, conidiation, and sclerotia formation in *A. flavus* [12]. Environmental factors including nutritional status and environmental stresses also play a role in the development and secondary metabolism in *Aspergillus* spp. [13,14].

Fungal cellular development and AFs biosynthesis are complicated processes involving many different types of transcription factors including the PHD family transcription factors, which are less abundant (about one kind) in the *A. flavus* genome (Figure S1) [15]. The PHD finger domain (plant homeodomain finger) was first discovered in the HAT3.1 protein from *Arabidopsis* [16]. PHD containing transcriptional factors have been found in various eukaryotic cells especially in plant and animal cells [17]. In recent years, a number of new PHD-finger protein homologs have been identified in different species. However, most putative PHD family members are still unrecognized and unidentified [18]. It was found that the PHD-finger protein DUET of *Arabidopsis* plays a regulatory role in chromosome organization and progression [19]. Some PHD proteins of mammals (such as UHRF1, ATX1, and ATX2) are essential for DNA methylation and the activity of histone methyl-transferase [20,21]. In addition, it was reported that the PHD-finger protein is closely related to the protein-protein interactions in human beings [22]. Since the PHD-finger protein homologs have potentially important functions in many species, it is important to study PHD family members in pathogenic fungi. As a PHD domain containing protein, Rum1 (regulator *U. maydis* 1) was found to play a critical role in the sporulation of *Ustilago maydis* and, according to its domains, it was speculated to function as a repressor in the process of transcription through chromatin structure modulation [23]. However, the biological function of PHD fingers containing Rum1 in the morphogenesis and mycotoxin biosynthesis of *Aspergillus* spp. has not been explored yet.

In view of the serious impacts of *A. flavus* on the safety and development of global society and economy and in the light of the putative critical role of the PHD transcriptional factor Rum1 in epigenetic regulation, it is of great importance to explore the biological function of Rum1 in *A. flavus*. Through sequence alignment with *rum1* sequence from *U. maydis*, we located a Rum1 ortholog (NCBI Gene bank No: AFLA_006240) in *A. flavus*. The study was carried out to explore the biological functions of Rum1 in the growth of mycelia, sporulation, sclerotial development, and aflatoxin production of *A. flavus*.

## 2. Results

### 2.1. Characterization of the PHD Transcription Factor Rum1 in A. flavus

**Figure 1:**
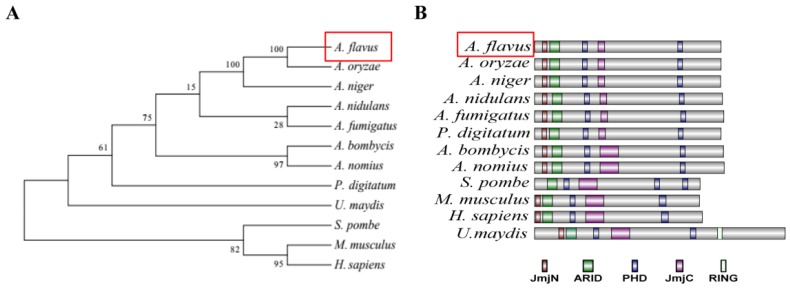
Characterization of the PHD transcription factor Rum1 in *A. flavus*. (**A**) Phylogenetic relationship of 12 Rum1 homologs from different species was analyzed with MEGA5.0. (**B**) The domain structure of Rum1 homologs was identified by SMART and were visualized using software IBS 1.0.

The homologs of 12 Rum1 proteins (from *A. flavus*, *A. niger*, *A. oryzae*, *A. nidulans*, *A. fumigatus*, *A. bombycis*, *A. nomius*, *P. digitatum*, *S. pombe*, *M. musculus*, *H. sapiens*, and *U. maydis*) were obtained from NCBI (http://www.ncbi.nlm.nih.gov), and the evolutionary relationship of these homologs was analyzed using MEGA5.0. The result showed that Rum1 in *Aspergillus* spp. was classified into a cluster in which the highest identity was found between *A. flavus* and *A. oryzae* (Ident 99%, Query cover 100%) and the lowest identity was found between *A. flavus* and *A. nidulans* (Ident 79%, Query cover 99%) (Figure 1A). Among non-*Aspergillus* spp., the highest identity was 76% (between *A. flavus* and *P*. *digitatum*, Query cover 98%) and the lowest identity was 30% (between *A. flavus* and *H. sapiens*, Query cover 77%). The protein domains in Rum1 were further analyzed with the use of software SMART, and IBS 1.0. A JmjN, an ARID, a JmjC, and two PHD fingers domains were found in almost all Rum1 homologs. Rum1 homolog from *S. pombe* has three PHD fingers domains, but lacks a JmjN domain, which is shown in Figure 1B. These results reflected that Rum1 is very conservative in fungi and mammals.

### 2.2. Construction of the Deleted Mutant (Δrum1) and Complemented Mutant (Δrum1-C)

**Figure 2:**
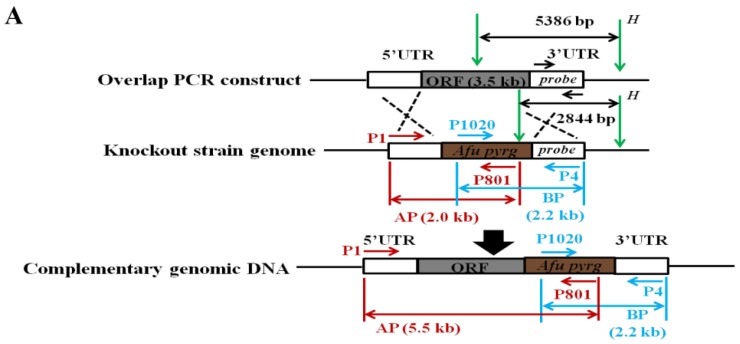

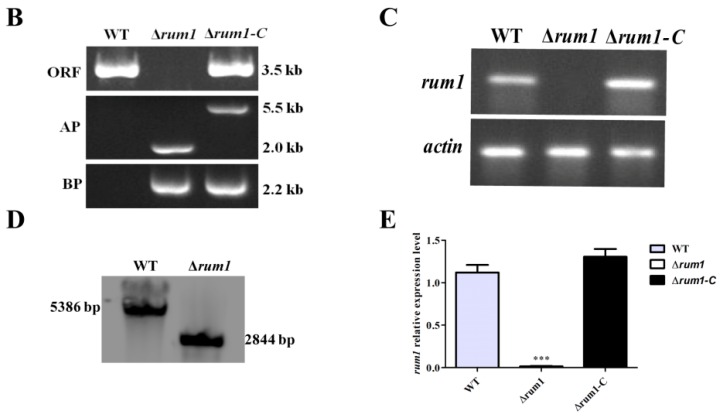
Strategy and confirmation of the mutant strains. (**A**) The scheme of *rum1* deletion and complementation strategy (*H*: *Hind*III, *probe*: the probe used in southern blot analysis). (**B**) Gene knockout and complemented strains were verified by PCR analysis. (The *rum1* ORF was confirmed by primers *rum1*-p9 and *rum1*-p10, AP fragment was confirmed by primers *rum1*-p1 and P801, and fragment BP was confirmed by primers P1020 and *rum1*-p4). (**C**) q-PCR verification of *rum1* gene deletion and complementation strains. The a*ctin* gene was used as an inner reference. (**D**) Southern blot analysis for ∆*rum1* mutant. Genomic DNA from above strains was digested with *Hind*III and hybridized with a 1.4 kb probe of the downstream region fragment of *rum1* (3′-UTR) (The probe fragment was amplified with primers *rum1*-p3 and *rum1*-p4), (**E**) q-PCR analysis of the expression level of *rum1* gene in ∆*rum1* WT and ∆*rum1-C* strains. *** represents significant difference (*p* < 0.001).

The *rum1* deletion mutant (∆*rum1*) was constructed through homologous recombination (Figure 2A) and was verified with PCR analysis through the amplification of *rum1* ORF, AP, and BP fragment using genomic DNA as a template in which a 3.5 kb ORF fragment was only amplified from WT and complementation (∆*rum1-C*) strains, a BP fragment (2.2 kb) was amplified from both ∆*rum1* and ∆*rum1-C* strains, a 2.0 kb AP fragment was amplified from ∆*rum1* strain, and a 5.5 kb AP fragment was amplified from ∆*rum1-C* strain (Figure 2B). Afterward, with cDNA as a template, a 220 bp DNA fragment from the ORF of *rum1* was amplified from WT and ∆*rum1-C* but not from ∆*rum1* mutant (Figure 2C). These results verified that the *rum1* gene had been deleted from the ∆*rum1* mutant and ∆*rum1-C* had been successfully constructed. The ∆*rum1* mutant was further verified with Southern blot analysis (Figure 2D). The result of q-PCR showed that no transcriptional activity of *rum1* could be detected in the ∆*rum1* mutant (Figure 2E), which confirmed that *rum1* had been deleted from the *rum1* mutant and the expression level of *rum1* recovered in ∆*rum1-C* was compared with the WT strain. Therefore, both the *rum1* deletion mutant (∆*rum1*) and complementation strain (∆*rum1-C*) were correctly constructed.

### 2.3. Rum1 Is Involved in Mycelium Growth and Conidiation

**Figure 3:**
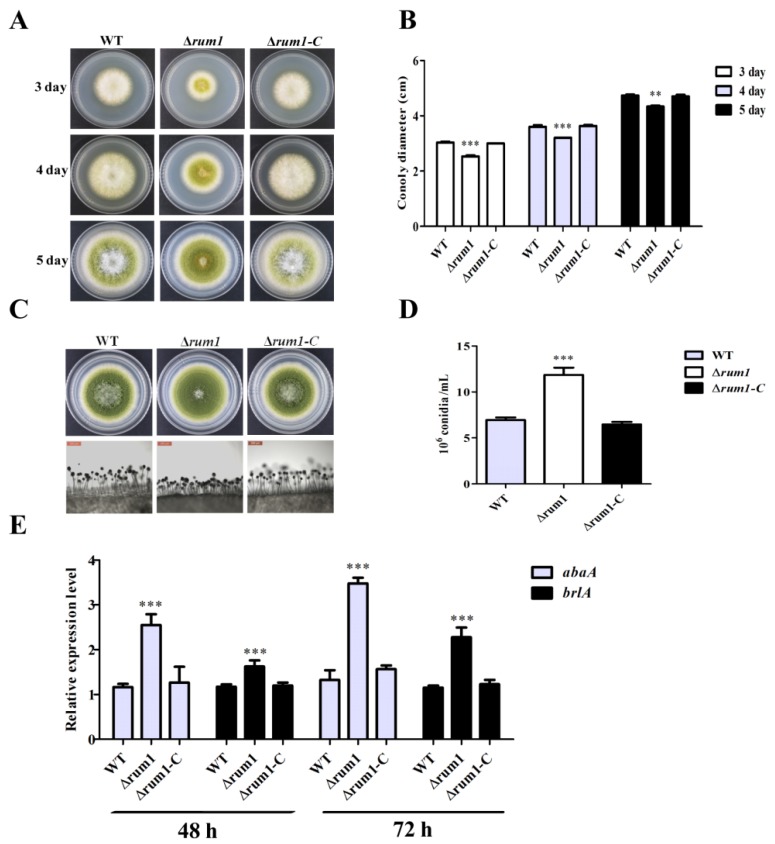
The roles of Rum1 in mycelium growth and conidiation in *A. flavus.* (**A**) The colonies of *A. flavus* strains grew on PDA medium at 29 °C. (**B**) The histogram of colony diameter calculated according to the result of A. (**C**) The conidiophores of *A. flavus* strains were observed under a microscope (20×). (**D**) The conidia of each plate were suspended with 5 mL of spore eluate. The conidia number of *A. flavus* strains in 1 ml spore eluate was calculated using hemocytometer. (**E**) The q-PCR analysis of *brlA* and *abaA* and the transcriptional factor genes for conidiation in the related *A. flavus* strains. ** and *** denote statistical significant levels of *p* < 0.01 and *p* < 0.001.

To research the role of Rum1 in the development of *A. flavus*, the WT, ∆*rum1*, and ∆*rum1-C* strains were incubated on PDA medium in the dark at 29 °C for 5 days, 37 °C for 5 days, and 42 °C for 10 days, respectively. The results showed that the ∆*rum1* mutant displayed no significant difference in colony growth compared to WT and ∆*rum1-C* strains under 37 °C and 42 °C (data not shown), but the diameter of the ∆*rum1* colony was significantly smaller than that of WT and the ∆*rum1-C* strains at 29 °C (Figure 3A,B). The results also revealed that the conidiation of the ∆*rum1* mutant was remarkably increased and the conidiophores of the ∆*rum1* mutant were remarkably shorter and denser than those of WT and the ∆*rum-C* strains (Figure 3C,D). Results of q-PCR analysis revealed that both *brlA* and *abaA* (the transcriptional factor genes for conidiation regulation) were significantly up-regulated in the ∆*rum1* mutant (Figure 3E). The above results suggested that Rum1 negatively regulates *A. flavus* conidiation through transcriptional factor genes *brlA* and *abaA.*

### 2.4. Rum1 Is Essential for Sclerotial Generation

**Figure 4:**
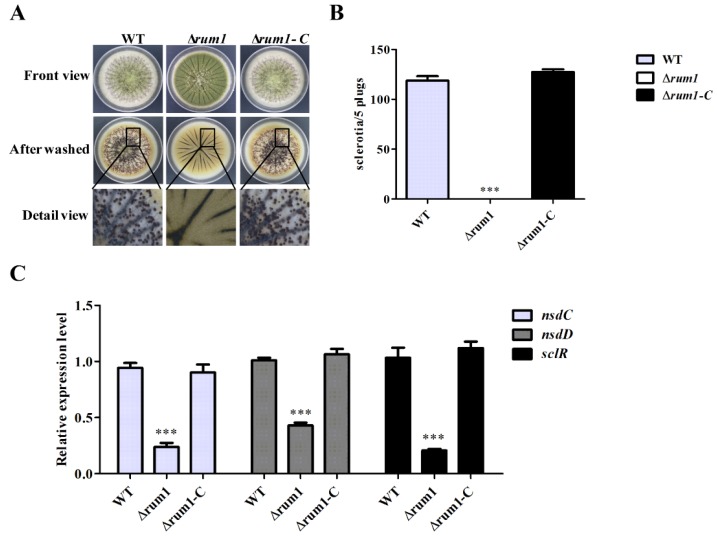
The role of Rum1 in sclerotia formation. (**A**) WT, ∆*rum1*, and ∆*rum1-C* strains were inoculated on WKM medium for 7 days and then the plates were sprayed with 75% ethanol to make sclerotia visible. (**B**) The histogram showing the amount of sclerotia according to (**A**), (**C**) Transcriptional expression levels of *nsdC*, *nsdD*, and *sclR*, which are the genes of positive regulators in sclerotia formation. *** represents significant difference (*p* < 0.001).

To explore the biological function of Rum1 in sclerotia formation, the WT, ∆*rum1*, and ∆*rum1-C* strains were incubated on the WKM and GMM medium (data not shown) at 37 °C in the dark for a week. We found that there was no sclerotia formed in the ∆*rum1* mutant strain compared to WT and ∆*rum1-C* strains on both media, which indicated that the deletion of *rum1* prominently declined the capability of sclerotia formation (Figure 4A,B). The results of the q-PCR analysis showed that the expression levels of *sclR*, *nsdC*, and *nsdD* genes (the positive regulators for sclerotia formation) were all transcriptionally down-regulated in the absence of Rum1 (Figure 4C). These results suggested that Rum1 positively regulates sclerotia formation through sclerotia formation regulators SclR, NsdC, and NsdD.

### 2.5. Rum1 Positively Regulates Aflatoxin Biosynthesis

**Figure 5:**
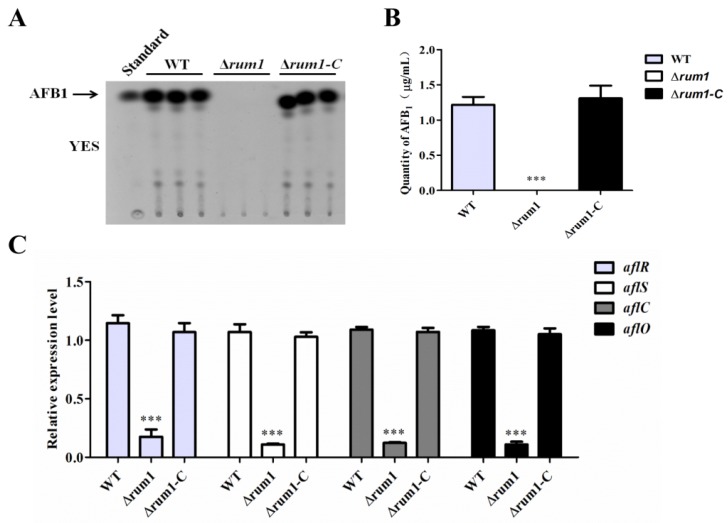
The role of Rum1 in aflatoxin biosynthesis. (**A**) AFB_1_ production of WT, ∆*rum1*, and ∆*rum1-C* strains was detected by Thin-Layer Chromatography (TLC). All strains were cultivated in liquid YES medium for 6 days at 29 °C in the dark (5 μL of each sample was allotted for the TLC analysis. Three lanes of the same strain represent biological repeats, respectively). (**B**) The quantity of AFB_1_ produced by related *A. flavus* strains was quantitated according to (**A**), (**C**) Transcriptional levels of aflatoxin biosynthesis related gene *aflR*, *aflS*, *aflC*, and *aflO* in the strains mentioned above. *** represents a significant difference (*p* < 0.001).

To examine the impact of Rum1 in the biosynthesis of AFB_1_, all related *A. flavus* strains were cultivated in liquid YES medium at 29 °C for 6 days and the samples were collected and prepared on the sixth day. Afterward, these samples were further analyzed by TLC. The results indicated that no AFB_1_ production could be found from the ∆*rum1* mutant compared to WT and ∆*rum1-C* strains (Figure 5A,B). We performed q-PCR to analyze the expression levels of AFB_1_ synthesis related genes (*aflR*, *aflS*, *aflC*, and *aflO*), which revealed that the expression levels of these genes were significantly decreased in the ∆*rum1* mutant compared to findings from the WT and ∆*rum1-C* strains (Figure 5C). These results revealed that Rum1 is crucial for AFB_1_ biosynthesis in *A. flavus*.

### 2.6. Rum1 is Involved in the Colonization of A. flavus to Crop Kernels

**Figure 6:**
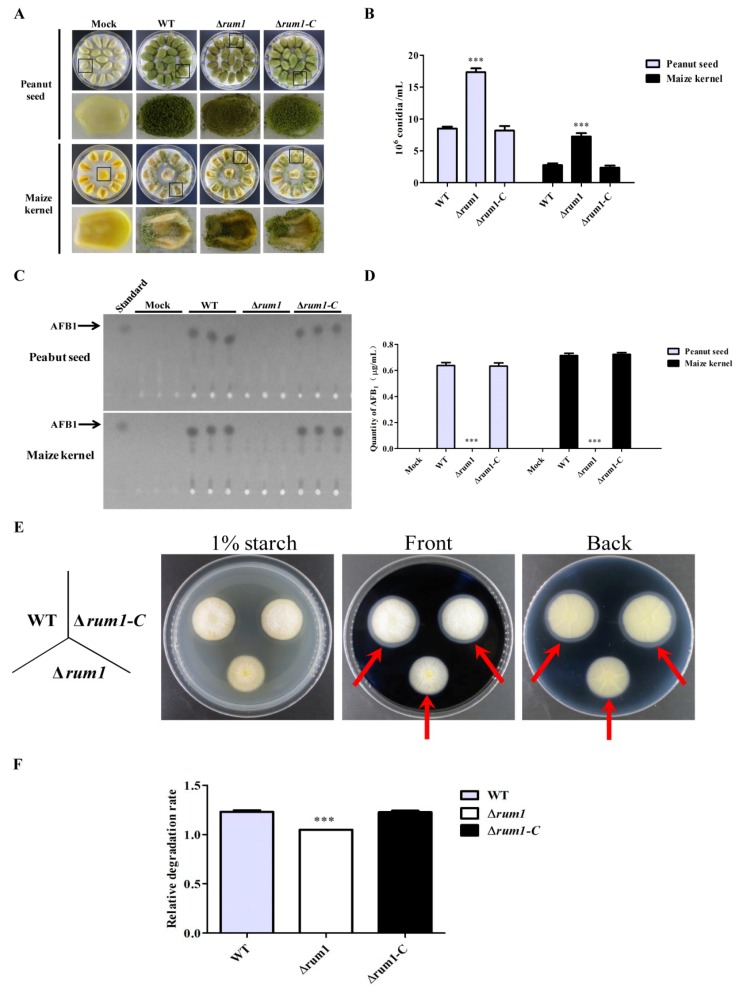
The role of Rum1 in *A. flavus* colonization. (**A**) Photographs presented the maize and peanut kernels colonized by WT, ∆*rum1*, and ∆*rum1-C* strains. (**B**) Conidia production of all the strains on seeds (The conidia of each plate were suspended with 5 mL of spore eluate and the conidia number of *A. flavus* strains in 1 mL spore eluate was calculated with hemocytometer). (**C**) The production of AFB_1_ from colonized kernels was detected by TLC (5 μL from each sample was loaded for the TLC analysis. Three lanes of the same strain represent three biological repeats, respectively). (**D**) Relative AFB_1_ production in (**C**) was quantitated. (**E**) Amylase activity was analyzed with iodine solution and 1% starch (Red arrows point to the degradation zones). (**F**) The relative degradation rate of starch of WT, ∆*rum1*, and ∆*rum1-C* strains, according to the results of (**E**). *** represents significant difference (*p* < 0.001).

To detect the biological function of Rum1 in colonization of *A. flavus*, maize and peanut kernels were incubated with conidia suspension of WT, ∆*rum1* mutant, and ∆*rum1-C* strains. The results (Figure 6A,B) showed that the conidiophore of the ∆*rum1* mutant was shorter when colonized on maize and peanut kernels. The ∆*rum1* mutant produced more conidia compared to WT and ∆*rum1-C* strains as the state found on the PDA medium (Figure 3C,D). The production of AFB_1_ from *A. flavus* colonized kernels was also explored, which showed that AFB_1_ production was severely restrained in the ∆*rum1* mutant (Figure 6C,D). These results demonstrated that Rum1 is crucial for conidiation and aflatoxin biosynthesis in the colonization process of *A. flavus* on crop kernels.

It was found that the ability of fungal colonization is closely related to changes in hydrolase activity [24,25]. To explore the biological function of Rum1 in *A. flavus* virulence, the role of Rum1 in the activity of amylase in *A. flavus* was explored with WT, ∆*rum1*, and ∆*rum1-C* strains. The results showed that the relative degradation rate of the ∆*rum1* mutant to starch was significantly smaller compared to that of WT and ∆*rum1-C* strains (*p* < 0.001) (Figure 6E,F. The relative degradation rate = diameter of degradation zone/diameter of colony). The findings suggested that Rum1 enhances the activity of amylase in fungi.

## 3. Discussion

The results of phylogenetic analysis in the study indicated that Rum1 is quite conserved from fungi to mammals, which suggested that Rum1 plays important and similar roles in fungal biological activity. The analysis of domain architectures also revealed that Rum1 is composed of a series of functional domains (including a JmjN, an ARID, a JmjC, and two PHD fingers domains). JmjC (Jumonji C) is a demethylase-related domain [26,27] and JmjN (Jumonji N) is always located adjacent to JmjC to form one functional unit [28,29]. ARID (AT-Rich Interacting Domain) is a DNA binding domain participating in the target-specific reaction in a Jumonji domain containing KDMs (histone-lysine demethylase) [30,31,32] and a host of PHD fingers have been characterized as a novel family of histone code readers recently [17,33]. Nevertheless, no reports about the biological function of the PHD transcription factor Rum1 in filamentous fungi were given until now. Our work revealed that Rum1 played important roles in the development, asexual reproduction, sclerotia formation, and aflatoxin biosynthesis in *A. flavus*.

This study showed that Rum1 improved the growth of mycelia at 29 °C, but negatively regulated conidiation by down-regulating the expression levels of *brlA* and *abaA* (Figure 3E). Both *abaA* and *brlA* are believed to play a key role in conidia development in which *brlA* is the first key transcription factor activated in the process of conidiation [34,35,36]. Afterward, *abaA* is activated by the *brlA* gene during the middle stage of conidiation [37]. In the process of *A. flavus* colonization of crops kernels (Figure 6A,B), the asexual reproduction of *A. flavus* was also obviously negatively regulated by Rum1. The reduction in asexual reproduction in pathogenic *Aspergillus* spp. in the presence of Rum1 lowers the chances of the plant pathogenic fungus to transfer from one host to the other. It is inferred in the study that Rum1 improves the formation of sclerotia by increasing the expression levels of *nsdC*, *nsdD*, and *sclR*. The genes *nsdC*, *nsdD*, and *sclR* were reported to promote sclerotial formation in *A. nidulans* and *A. oryzae* [38,39]. It was found recently that the sexual reproduction structure-ascocarps were embedded in the sclerotia of *A. flavus* [40], which illuminated that the lack of Rum1 led to the abortion of sclerotia formation in the ∆*rum1* mutant in the study. This could mean that Rum1 might play an important role in promoting the genetic variation of filamentous fungi by sexual reproduction to improve environmental adaptability of the plant pathogenic fungus. Sclerotia are commonly considered survival structures against unfavorable conditions. Therefore, the presence of Rum1 is clearly in favor of the survival of the plant pathogenic fungus against an adverse environment.

In YES liquid medium (also in PDB medium, data not shown) and crop colonization models (maize and peanut), the results showed that Rum1 dramatically enhanced the AFB_1_ biosynthesis by up-regulating the expression levels of aflatoxin biosynthesis structural genes *aflC* and *aflO* as well as regulatory genes *aflR* and *aflS* (Figure 5C). As a 47 kDa zinc-finger transcriptional factor, AflR is a positive regulator for aflatoxin synthesis and the transcription of most structural genes in the aflatoxin gene cluster require the positive regulation of AflR [10]. AflS, regulated by AflR, is indispensable for aflatoxin bio-synthesis [40]. In the *aflS* knockout mutants, the expression levels of some structural genes (e.g., *aflC*, *aflD*, *aflM*, and *aflP*) in aflatoxin pathway were found to have a five-fold to 20-fold reduction. In addition, genes known as *aflC* and *aflO* catalyze the chemical reaction of acetate to norsolorinic acid and DMST to ST in the biosynthesis pathway of AFB_1_, respectively [41]. In this study, it was found that the expression level of *aflR* dramatically decreased by approximately five-fold in ∆*rum1* mutant (*p* < 0.001) (Figure 5C). Meanwhile, the possible responsible elements (RE) of the *aflR* promoter were predicted with MEME online (http://meme-suite.org/tools/meme), which revealed that Rum1 might bind to the possible RE on the promoter of *aflR* to regulate the synthesis of aflatoxin (Figure S2). Above results suggested that Rum1 triggered the biosynthesis of aflatoxin by activating the aflatoxin biosynthesis-relevant gene cluster in *A. flavus*.

The role of Rum1 in the colonization and virulence of *A. flavus* on crop kernels is critical and complicated. In the study, Rum1 was found to significantly repress conidiation and to initiate sclerotial development (Figure 4 and Figure 6, *p* < 0.001), which seemed that Rum1 attempted to initiate sexual reproduction by depressing asexual activity to some degree in the colonization of *A. flavus* to crops. Similar to the results from YES liquid medium (Figure 5) and PDB medium (data no shown), the absence of Rum1 deprived the plant pathogenic fungus of aflatoxin biosynthesis ability in the colonization of *A. flavus* on crops (Figure 6), which revealed that Rum1 is an important virulence factor in the fungus. Plant pathogenic fungi might encounter some stresses inside host crops. The role of Rum1 under the stresses was also explored in this study. No statistical significance was found between the inhibition rate of the ∆*rum1* mutant and the inhibition rates of WT and the ∆*rum1-C* strains under stresses tested in the study (Figure S3), which suggested that Rum1 didn’t participate in the stress responses of *A. flavus*. It was reported that the capacity of fungal colonization is bound up with the changes of the hydrolytic enzymes’ activity [24,25]. The tests on hydrolytic enzymes in the study suggested that Rum1 may affect colonization of plant pathogenic fungi by increasing the activity of amylase in fungi (Figure 6E,F). In summary, the results of our study suggested that Rum1 might be involved in the colonization and virulence of *A. flavus* on crop kernels through its roles in fungal development, secondary metabolism, and in the activity of some hydrolytic enzymes.

## 4. Conclusions

In conclusion, the PHD transcription factor Rum1 of *A. flavus* is deeply involved in the morphogenesis and AFs biosynthesis of the fungus. Our findings provided meaningful information that could improve our understanding of the roles of Rum1 in the regulatory mechanisms of secondary metabolism and fungal morphogenesis in the plant pathogenic fungus, which might lay a foundation for developing new control strategies against AFs producing fungus.

## 5. Materials and Methods

### 5.1. Fungal Strains and Primers

All *A. flavus* strains used in the study were listed in Table 1 and the primers were shown in Table 2 and Table 3. The Wickerham medium (WKM, 2 g/L yeast extract, 3 g/L peptone, 5 g/L cornteep solids, 2 g/L dextrose, 30 g/L sucrose, 2 g/L NaNO_3_, 1 g/L K_2_HPO_4_•3H_2_O, 0.5 g/L MgSO_4_•7H_2_O, 0.2 g/L KCl, 0.1 g/L FeSO_4_•7H_2_O), YES medium (20 g/L yeast extract, 150 g/L sucrose, 1 g/L MgSO_4_•7H_2_O), potato dextrose agar (PDA, 39 g/L, BDDifco, Franklin, NJ, USA), potato dextrose broth (PDB, 24 g/L, BDDifco, Franklin, NJ, USA), and GMM medium (10 g/L dextrose, 10 mM/L ammonium tartrate, 1.52 g/L KH_2_PO_4_, 0.52 g/L MgSO_4_•7H_2_O, 0.52 g/L KCl, and 1 mL trace elements) were prepared for fungal cultivation in this study. For solid medium, agar was added at 15 g/L. For the auxotrophic marker (*pyrG*-), the medium was supplemented with 1 mg/mL uracil and 1 mg/mL uridine [12].

### 5.2. Phylogenetic Analysis

Rum1 orthologs from *A. flavus*, *A. niger*, *A. nidulans*, *A. oryzae*, *A. fumigatus*, *A. bombycis*, *A. nomius*, *P. digitatum*, *S. pombe*, *M. musculus*, *H. sapiens*, and *U. maydis* were obtained from the National Center for Biotechnology Information (NCBI, https://www.ncbi.nlm.nih.gov/). The phylogenetic tree was constructed for the above 12 Rum1 orthologs by MEGA 5.0 with an algorithm of 1000 times Neighboring comparison. In the study, SMART (http://smart.embl-heidelberg.de/smart/set_mode.cgi?NORMAL=1) was used to identify the domains in Rum1 and the framework of domains was mapped with software IBS 1.0 (Lab of Cell Dynamics, and Lab of Nanobiology, University of Science & Technology of China, Hefei, Anhui, China) [42].

### 5.3. Mutant Strains Construction

The ∆*rum1* mutants were prepared by a homologous recombination approach [39]. Three fragments (1200 bp 5′-UTR of *rum1*, 1411 bp 3′-UTR and 1890 bp *pyrG*) were amplified and overlapped by Overlap PCR and the primers used in the Overlap PCR were shown in Table 2. Afterward, the transformation of protoplasts (CA14 Δ*ku70* Δ*pyrG*) was carried out, which was mediated by PEG (polyethylene glycol) [12]. The *pyrG* prototroph strain (∆*rum1*, Table 1) was further tested with the Southern blot method and PCR analysis (related primers shown in Table 2).

The complementation strains for the ∆*rum1* mutants were constructed following the method provided by Yang [43]. First, the *rum1* fragment amplified from *A. flavus* with *rum1*-p7/*rum1*-p8 primers were transformed into the protoplasts of ∆*rum1* with 2 mg/mL 5-FOA (5-fluoroorotic acid) to replace *pyrG* [44]. PCR analysis was performed to confirm that the *pyrg* gene was removed from the first step of complemented strains (Figure S4). Lastly, the *Pyrg* gene was inserted behind the *rum1* to produce the *pyrG* prototroph complementation strains (∆*rum1-C*) by using a homologous recombination. The complementation strain was tested by using PCR analysis. Both of the verified ∆*rum1* mutant and ∆*rum1-C* strains were further confirmed by a q-PCR assay with an *actin* gene as the inner reference. The primers used in the analysis are listed in Table 3.

### 5.4. Real-Time Quantitative Reverse Transcription PCR

The analysis was implemented according to the protocol used by Nie [45]. RNA was extracted from 0.1 g of a paper towel dried mycelium using an RNA isolation kit (Promega, Madison, WI, USA). Then, the synthesis of first-strand cDNA from 3 μg RNA was performed using the Revert Aid First-strand cDNA Synthesis kit (TransGen Biotech, Beijing, China). The SYBR Green Supermix (Takara, Waltham, MA, USA) was used for the qRT-PCR reaction with the PikoReal 96 Real-time PCR system. All primers used in the assay were listed in Table 3. All experiments were repeated three times.

### 5.5. Morphological Analysis

The analysis was carried out according to the method used by Lan [46]. The WT, ∆*rum1*, and ∆*rum1-C* strains were incubated onto PDA medium in the dark at 37 °C and the diameter of each colony was measured after 5 days. Conidia were homogenized in 5 mL water solution (0.05% Tween-20) and the conidia number was counted haemocytometrically from 1 mL conidia mixture. For sclerotia production analysis, sclerotia inducing WKM medium was prepared and all cultures were grown at 37 °C for a week in the dark. Lastly, 75% ethanol was used to spray the surface of each plate to wash conidia away. Then sclerotia were collected and the number of sclerotia was counted with the Asana microscope. Above experiments were completed with three repetitions.

### 5.6. Aflatoxin Analysis

The WT, ∆*rum1*, and ∆*rum1-C* strains were incubated into 15 mL PDB and YES media at 29 °C in the dark for 6 days, respectively. Then AFB_1_ was extracted from the culture, which was previously described by Yang [43]. AFB_1_ production was analyzed with thin layer chromatography (TLC) (5 μL for each sample was used for the TLC analysis) and examined under a UV light. The pictures of the TLC plates were inverted by the PS software to get clearer data. The experiments were repeated three times.

### 5.7. Stress Assays

The WT, the ∆*rum1*, and the ∆*rum1-C* strains were inoculated onto PDA agar with hyperosmotic stress mediators (1 M NaCl, 1 M KCl, or 1.2 M D-Sorbitol), oxidative stress agent (5–10 mM H_2_O_2_), DNA damaging agent (0.02% MMS (Methyl methanesulfonate)), and cell wall stress agents (200 μg/mL CFW (Calcofluor white) or 300 μg/mL CR (Congo red)) at 37 °C in the dark for 3 days, respectively. To analyze the role of Rum1 in stress response of *A. flavus*, the relative inhibition rates were calculated, according to the formula listed in the brackets (diameter of colony without inhibitor—diameter of colony with inhibitor/diameter of colony without inhibitor). The experiments were performed in three repetitions.

### 5.8. Crop Kernels Colonization Assays

To explore the role of Rum1 in crop colonization, the assay was carried out following the methods used by Lan with minor modification [46]. Kernels were sterilized with 0.05% sodium hypochlorite and inoculated with conidia at 29 °C for 5 days. Afterward, the colonized kernels were collected in 50 mL Falcon tubes, mixed with 10 mL sterile 0.05% Tween 80, and followed by 2 min in a vortex state to release the spores. Additionally, 1 mL aliquot of spore suspension was diluted and counted haemocytometrically. A total of 15 mL of chloroform was added to each Falcon tube for AFB_1_ extraction and these tubes were placed on the 29 °C shaker and shaken at 180 r/min for 30 min. The lower chloroform layer (10 mL) was collected and dried by airing. After re-dissolving in 1 mL chloroform, 5 µL of each sample was allotted for the TLC assay. The experiments were repeated three times.

### 5.9. The Analysis on the Activity of Amylase

The activity of amylase was analyzed according to the protocol used by Li [47]. The WT, the ∆*rum1*, and the ∆*rum1-C* strains were incubated on the amylase screening medium (10 g/L peptone, 10 g/L yeast extract, 1% soluble starch, 15 g/L Agar) in the dark at 29 °C for 3 days. Iodine solution was added evenly in the plate and the diameter of the degradation zone was measured. The relative degradation rate equals the diameter of degradation zone/colony diameter.

### 5.10. Statistical Analysis

In this study, all data were presented with the means ± SD (standard deviation). The significant differences (statistical significances) among groups were calculated with ANOVA and LSD (least significant difference) tests. The analysis of statistical and significance was implemented with the software GraphPad Prism5 (La Jolla, CA, USA) and the difference is regarded to be statistically significant when *p* < 0.05.

## Figures and Tables

**Table 1:** Fungal strains used in the study.

Strain	Genotype Description	Reference
*A. flavus* CA14	∆*ku70*, ∆*pyrG*	purchased from FGSC
wild-type (WT)	∆*ku70*, ∆*pyrG*::*AfpyrG*	This study
∆*rum1*	∆*ku70*, ∆*rum1*::*AfpyrG*	This study
∆*rum1-C*	∆*ku70*, ∆*rum1*::*AfpyrG*, *rum1*::*AfpyrG*	This study

**Table 2:** Primers used for the strain construction in this study.

Primer Name	Sequence (5′-3′)	Fragment
*rum1*-p1	GGCACGAGCTATTAGTGATATTAGTCGAGTCCGA	5′UTR of *rum1*
*rum1*-p2	CAAGTGAGCCGACCGATTGAGGGAAGTAGT	
*rum1*-p3	TCCCTATCAACAAATTGGCGCTTCATGGGTTC	3′UTR of *rum1*
*rum1*-p4	TGGATTCCTTCGGGGGCTAGTTTGCATC	
*rum1*-p5	ACTACTTCCCTCAATCGGTCGGCTCACTT	*A. fumigatus pyrG*
	GGCCTCAAACAATGCTCTTCACCC	
*rum1*-p6	GAACCCATGAAGCGCCAATTTGTTGATA	
	GGGAGTCTGAGAGGAGGCACTGATGC	
*rum1*-p7	GACCTGTGAAGATGCTTGGTAGAGCTATTTCAG	Nesting primers
*rum1*-p8	TATCTCATTGGACTGGACCCTGAGCGGGA	
*rum1*-p9	CAACTCGACTGGCGGACAGCCT	A fragment from *rum1*
*rum1*-p10	TCATTTGCCGGAGAATATGTTCCAGTCCTTC	
P801	CAGGAGTTCTCGGGTTGTCG	*A. fumigatus pyrG*
P1020	CAGAGTATGCGGCAAGTCA	
*rum*-p3 + *pyrg*-F	CTTCATCGCGAGATAACACCCCCGATGG	5′UTR of Δ*rum1-C*
*rum*-p3 + *pyrg*-R	GGGTGAAGAGCATTGTTTGAGGCCCCATG	
*pyrg*-F	GCCTCAAACAATGCTCTTCACCC	*A. fumigatus pyrG*
*pyrg*-R	GTCTGAGAGGAGGCACTGATGC	
*rum*-p4 *+ pyrg*-F	GCATCAGTGCCTCCTCTCAGACAGATTCTT	3′UTR of Δ*rum1-C*
	GCCTTGCGCATTCATGACAAC	

**Table 3:** Primers for q-PCR analysis in the study.

Gene	Forward Sequences (5′-3′)	Reverse Sequences (3′-5′)
*rum1*	CTTGATGCATCTCTCTTT	CTTCCAGAGCCTCATTA
	AGCTCTCCACGGTTC	GCATGTGTGTTCTCC
*brlA*	GCCTCCAGCGTCAACCTTC	TCTCTTCAAATGCTCTTGCCTC
*abaA*	TCTTCGGTTGATGGATGATTTC	CCGTTGGGAGGCTGGGT
*nsdC*	GCCAGACTTGCCAATCAC	CATCCACCTTGCCCTTTA
*nsdD*	GGACTTGCGGGTCGTGCTA	AGAACGCTGGGTCTGGTGC
*sclR*	CAATGAGCCTATGGGAGTGG	ATCTTCGCCCGAGTGGTT
*aflR*	AAAGCACCCTGTCTTCCCTAAC	GAAGAGGTGGGTCAGTGTTTGTAG
*aflS*	CGAGTCGCTCAGGCGCTCAA	GCTCAGACTGACCGCCGCTC
*aflC*	GTGGTGGTTGCCAATGCG	CTGAAACAGTAGGACGGGAGC
*aflO*	GATTGGGATGTGGTCATGCGATT	GCCTGGGTCCGAAGAATGC
*actin*	ACGGTGTCGTCACAAACTGG	CGGTTGGACTTAGGGTTGATAG

## Supplementary Materials

The following are available online at http://www.mdpi.com/2072-6651/10/7/301/s1, Figure S1: Classes of transcriptional factors in *A. flavus* [15], Figure S2: Predicted RE of Rum1 in transcriptional factor genes of *A. flavus*, Figure S3: Growth of WT, the ∆*rum1* strains, and the ∆*rum1-C* strains under multiple stresses, Figure S4. Strategy and confirmation of the complemented strain.
