# Supplementary material for: The PHD Transcription Factor Rum1 Regulates Morphogenesis and Aflatoxin Biosynthesis in Aspergillus flavus
Source: Toxins (Basel). 2018 Jul 20;10(7):301. doi: 10.3390/toxins10070301 (PMC6070901; doi:10.3390/toxins10070301)
Supplement: Supplementary file 1 [file toxins-10-00301-s001.pdf]

Supplementary Materials: The PHD Transcription Factor Rum1 Regulates Morphogenesis and Aflatoxin Biosynthesis in *Aspergillus flavus*

Yule Hu, Guang Yang, Danping Zhang, Yaju Liu, Yu Li, Guanglan Lin, Zhiqiang Guo, Shihua Wang and Zhenhong Zhuang

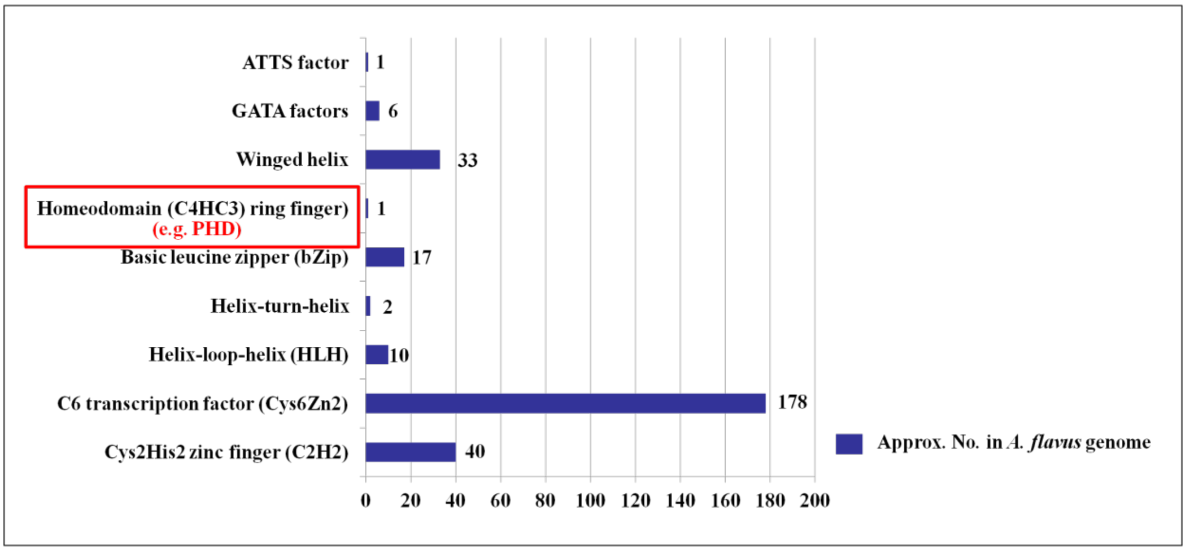

Figure S1. Classes of transcriptional factors in *A. flavus* [15]. Example of Homeodomain (C4HC3) ring finger: PHD.

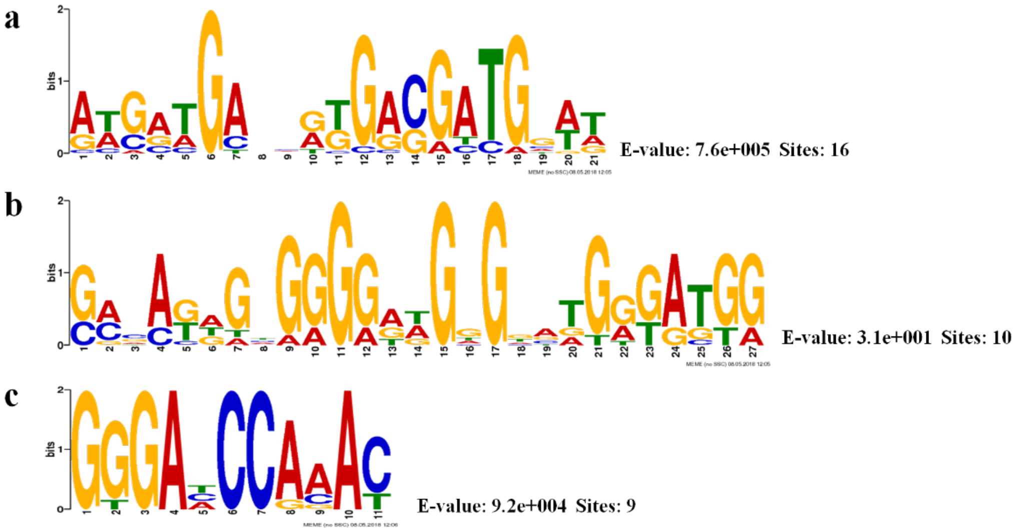

Figure S2. Predicted RE of Rum1 in transcriptional factor genes of *A. flavus*. (genes including: AFLA_139360 *aflR* [*Aspergillus flavus* NRRL3357], AFLA_139340 *aflS* [*Aspergillus flavus* NRRL3357], AFLA_139410 *aflC* [*Aspergillus flavus* NRRL3357], AFLA_139220 *aflO* [*Aspergillus flavus* NRRL3357], AFLA_082850 *brlA* [*Aspergillus flavus* NRRL3357], AFLA_029620 *abaA* [*Aspergillus flavus* NRRL3357], AFLA_020210 *nsdD* [*Aspergillus flavus* NRRL3357]. MEME website address: http://meme-suite.org/tools/meme. **(a)** E-value: 7.6e+005, Sites: 16. **(b)** E-value: 3.1e+001, Sites: 10. **(c)** E-value: 9.2e+004, Sites: 9.

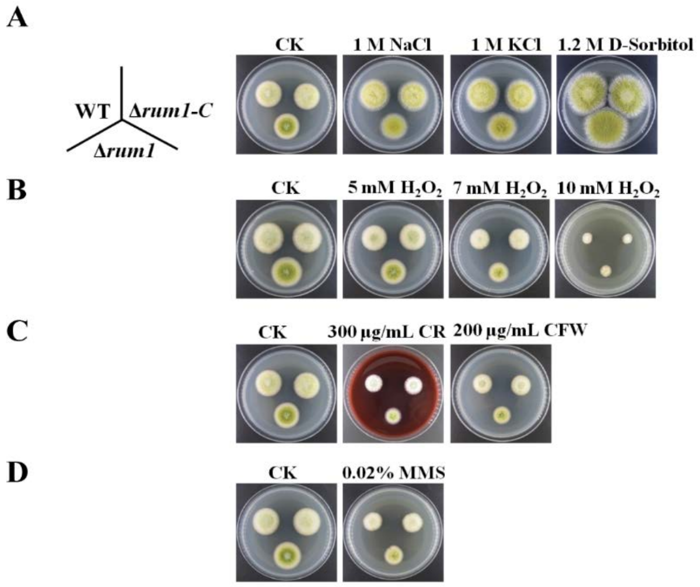

Figure S3. Growth of WT, Δ*rum1* and Δ*rum1-C* strains under multiple stresses. (**A**) Phenotype of WT, Δ*rum1* and Δ*rum1-C* strains under hyperosmotic stress (1 M NaCl, KCl, and 1.2 M D-Sorbitol); (**B**) Morphology of different strains under oxidative stress (5-10 mM H_2O_2); (**C**) Colony phenotype of different strains cell wall stress (300 μg/mL CR and 200 μg/mL CFW); (**D**) Phenotype of different strains under DNA damaging agent stress (0.02% MMS).

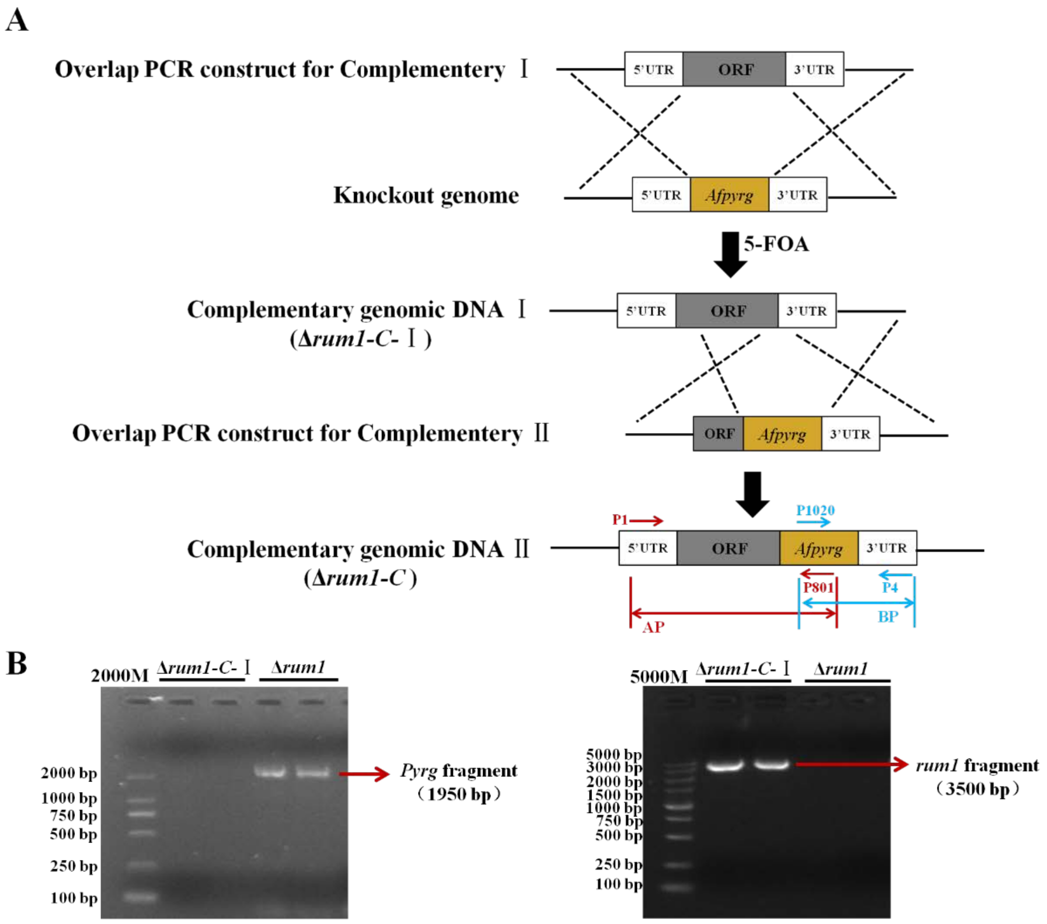

Figure S4. Strategy and confirmation of the complemented strain. **(A)** The scheme for *rum1* complement strategy. (**B**) PCR analysis was performed to confirm *rum1* complemented strains in the first step. ("Δ*rum1-C-* Ⅰ"represents the first step of complemented strain, "Δ*rum1*" represents *rum1*

gene knockout mutant. *Pyrg* fragment was confirmed by primers *rum1*-p5 and *rum1*-p6. *rum1* fragment was confirmed by primers *rum1*-p9 and *rum1*-p10. Two lanes of the same strain represent two repeats, respectively).
